# Assessing fidelity to family-based treatment: an exploratory examination of expert, therapist, parent, and peer ratings

**DOI:** 10.1186/s40337-020-00366-5

**Published:** 2021-01-14

**Authors:** Jennifer Couturier, Melissa Kimber, Melanie Barwick, Gail McVey, Sheri Findlay, Cheryl Webb, Alison Niccols, James Lock

**Affiliations:** 1 Department of Psychiatry & Behavioural Neurosciences, McMaster University, Hamilton, Canada; 2 Offord Centre for Child Studies, McMaster University, Hamilton, Canada; 3 Research Institute, The Hospital for Sick Children, Toronto, Canada; 4 Toronto General Hospital Research Institute, University Health Network, Toronto, Canada; 5 Department of Pediatrics, McMaster University, Hamilton, Canada; 6 Department of Psychiatry & Neurosciences, Stanford University, Stanford, USA

**Keywords:** Fidelity, Family-based therapy, Anorexia nervosa, Adherence, Adolescent

## Abstract

**Introduction:**

Fidelity is an essential component for evaluating the clinical and implementation outcomes related to delivery of evidence-based practices (EBPs). Effective measurement of fidelity requires clinical buy-in, and as such, requires a process that is not burdensome for clinicians and managers. As part of a larger implementation study, we examined fidelity to Family-Based Treatment (FBT) measured by several different raters including an expert, a peer, therapists themselves, and parents, with a goal of determining a pragmatic, reliable and efficient method to capture treatment fidelity to FBT.

**Methods:**

Each therapist audio-recorded at least one FBT case and submitted recordings from session 1, 2, and 3 from phase 1, plus one additional session from phase 1, two sessions from phase 2, and one session from phase 3. These submitted files were rated by an expert and a peer rater using a validated FBT fidelity measure. As well, therapists and parents rated fidelity immediately following each session and submitted ratings to the research team. Inter-observer reliability was calculated for each item using the intraclass correlation coefficient (ICC), comparing the expert ratings to ratings from each of the other raters (parents, therapists, and peer). Mean scale scores were compared using repeated measures ANOVA.

**Results:**

Intraclass correlation coefficients revealed that agreement was the best between expert and peer, with excellent, good, or fair agreement in 7 of 13 items from session 1, 2 and 3. There were only four such values when comparing expert to parent agreement, and two such values comparing expert to therapist ratings. The rest of the ICC values indicated poor agreement. Scale level analysis indicated that expert fidelity ratings for phase 1 treatment sessions scores were significantly higher than the peer ratings and, that parent fidelity ratings tended to be significantly higher than the other raters across all three treatment phases. There were no significant differences between expert and therapist mean scores.

**Conclusions:**

There may be challenges inherent in parents rating fidelity accurately. Peer rating or therapist self-rating may be considered pragmatic, efficient, and reliable approaches to fidelity assessment for real-world clinical settings.

## Plain English summary

This is the first study to examine fidelity to Family Based Treatment for adolescents with eating disorders from the perspectives of multiple raters including expert, therapist, parent, and peer. Although these data are exploratory in nature, the peer rater demonstrated the highest concordance with the expert rater, while therapist raters and parent raters demonstrated low concordance with the expert on an item level. On a scale level, parents rated significantly higher than experts, while there were no significant differences between therapists and experts. These data suggest that peer ratings or therapist self-ratings might present practical, efficient, and reliable approaches to fidelity assessment for real-world clinical settings.

## Introduction

Implementing new evidence-based treatments (EBTs) in routine clinical practice is fraught with challenges, and evaluation of such implementation is even more difficult. Growing evidence from the field of implementation science points to the importance of evaluating both clinical and implementation outcomes; the latter includes practitioner fidelity to the treatment being implemented [1–3]. Treatment fidelity can be defined as the extent to which a practitioner delivers the EBT as intended by its developers, and consists of three elements: 1) adherence, or the extent to which a treatment and its core components are delivered; 2) competence, or the quality or skill with which a treatment is delivered; and 3) differentiation, or the degree to which the treatment differs from other interventions [4, 5]. The assessment of EBT fidelity is more common in academic settings, with evidence suggesting that fidelity is positively associated with clinical outcomes [6]. However, assessment of fidelity in real-world practice settings remains challenging and has lagged behind [7]. Evidence suggests that clinicians view the time and commitment required for regular fidelity checks too onerous in light of ever-increasing clinical and administrative demands [8]. The development of fidelity measures that are pragmatic, efficient and reliable may help surmount these barriers.

### Treatment Fidelity and outcomes

There is evidence that outcomes can be affected by treatment fidelity. A recent systematic review and meta-analysis indicated that therapist adherence was significantly and positively associated with favourable patient outcomes, except when client-informants rated intervention adherence [6]. In addition, a longitudinal evaluation of therapist fidelity to the Family-Check-Up (a behaviourally-based, parent management training program) indicated a significant, linear decline in therapist fidelity over time; steeper declines in therapist fidelity were associated with significantly higher scores of parent-reported problem behaviours in their children [9]. Notably, Schoenwald and colleagues [10] demonstrated that expert consultation improved clinician fidelity to the treatment model and, in turn, child outcomes [10]. In particular, the competence of the consultant was more important than the supportive alliance between consultant and clinician, resulting in increased clinician fidelity and improved youth outcomes in a study examining the implementation of multisystemic therapy in community-based settings for youth behaviour problems. This evidence suggests that ongoing expert consultation in the treatment model may be facilitative of therapist fidelity over the long term.

### Family-based treatment

Family-Based Treatment is a first-line treatment for child and adolescent eating disorders based on several high-quality primary studies [11–13] and a meta-analysis [14]. Despite strong evidence of effectiveness however, one Ontario study found that few therapists reported practicing FBT with adherence to the manual [15]. Several determinant factors were found to enhance or hinder the uptake of FBT including therapist motivation and confidence about FBT core elements, team and administrator buy-in to the FBT model, feasibility of fidelity monitoring processes, as well as previous training [15, 16]. In addition, Kosmerly and colleagues [17] found that over a third of clinicians surveyed reported delivering FBT that deviated quite substantially from the manual, due to caseload and anxiety. These findings are alarming given that delivery of core elements of the FBT model have been found to be significantly and positively associated with improvement in eating disorder symptomatology, particularly weight gain [18]. Contradictory evidence has emerged in a recent large study that did not find a relationship between weight as a primary outcome and therapist adherence to FBT when fidelity was rated by trained observers [19]. Therapist adherence to FBT has also been shown to decrease over the three phases of treatment and to be higher for more behaviourally-based interventions of FBT as opposed to more process-related items [19, 20] further adding to the complexity of the relationship between fidelity and clinical outcome.

When considering real-world implementation of FBT, expert and peer fidelity ratings can be too costly for service providers. To our knowledge, no study has considered the tenability of implementing parent-rated or therapist self-rated FBT fidelity to evaluate therapist adherence to the FBT model, although these methods have been used in other areas of mental health EBT implementation [21–24]. The present study explored the implementation of FBT in routine care, with treatment fidelity among the handful of implementation outcomes measured. We examined fidelity ratings provided by several different raters including an expert rater, therapist self-rater, parent rater, and peer rater with hopes of determining a pragmatic, reliable and efficient method to capture treatment fidelity to FBT.

## Methods

### Design

Data for this study came from a larger, multi-site (*n* = 4), mixed method, pre-post implementation study that used an evidence-informed implementation approach to improve capacity for Ontario-based therapists to deliver FBT to children and adolescents diagnosed with eating disorders [25]. As such, the context for this FBT implementation was research-initiated and supported.

Briefly, informed by the Active Implementation Frameworks which involves four phases of implementation and the use of implementation teams, [26] the larger study purposefully recruited therapists, physicians and administrators in four Ontario-based pediatric eating disorder programs to undergo training and clinical consultation in the FBT model, and explored implementation processes and therapist experiences of clinical consultation [25]. Working with the research team to support the sustainability of FBT at each site, each of the participating organizations was asked to identify an implementation team that consisted of an administrator/manager, a lead therapist and a medical practitioner who would be charged with supporting FBT training, supervision, implementation and research processes. Each implementation team was asked to identify therapists in their program who were most appropriate and willing to undergo training in the FBT model, receive clinical consultation with respect to their FBT practice, and willing to participate in the study’s research processes. Our findings with respect to clinical consultation and implementation consultation experiences are reported on elsewhere [27, 28]. We did not limit the number of therapists who could participate in the FBT training and implementation endeavour, but implementation teams were limited to nominating therapists who would ultimately deliver FBT to children and adolescents diagnosed with anorexia nervosa (AN) or bulimia nervosa (BN) during and after the study.

### The treatment model

FBT is an outpatient, intensive treatment in which the family is the primary resource to re-nourish the affected child [29]. FBT involves three phases of treatment over 9 to 12 months. The first phase focuses on helping the family to restore the child’s weight and interrupt disordered eating behavior. The second phase involves the transition of control over eating behavior back to the adolescent. The third and final phase addresses developmental issues such as physical development, peers and dating, and separation and individuation. FBT requires the therapist to weigh the patient, graph the weight and reveal the weight to the patient and family at each session. The therapist coaches the parent(s), who are charged with making decisions about nutrition and exercise without consultation from a dietician [29].

### Study procedures and data collection

The administrators, lead therapists, medical practitioners and selected therapists from each participating organization participated in a two-day FBT training workshop provided by an international FBT expert (JL) that was organized and attended by members of the research team (JC, MK, TW, SF, CW). Following the training workshop, all lead and selected therapists (*n* = 8) were asked to: (1) treat at least one adolescent patient presenting with AN or BN using the FBT model; (2) rate their own fidelity and obtain parent fidelity ratings of the FBT model at each session; and (3) participate in monthly telephone clinical consultation with an FBT expert (JC; 1 hour per call). Implementation consultation was also provided to implementation teams on a monthly basis. Details on our implementation approach have been previously published [25].

Adolescent patients and their families provided consent to be included in the study, as did therapists, administrators and medical practitioners.

### Fidelity measurement

Sessions were audio-recorded, shared with the research team through an online encrypted system, and reviewed for fidelity to the FBT model by the local FBT expert (JC) and by the peer rater (MK). The local FBT expert is a certified FBT therapist who has received extensive training from an international FBT expert (JL). The peer rater (MK) was a master’s level social worker with clinical experience in child and adolescent mental health, and doctorate level researcher who had basic training in FBT. No additional training was provided to the peer rater in terms of fidelity rating, as the purpose of our study was to see how a peer rater in routine clinical practice would be able to rate the FBT session without any extensive training in FBT or in FBT adherence rating.

Fidelity of each audio-recorded therapy session was rated by the local expert (JC) and peer (MK) using a validated FBT adherence measure used in other studies [19, 20]. The FBT fidelity measure was further developed subsequent to our study to include therapist competence in addition to therapist adherence, and was renamed the Family Therapy Fidelity and Adherence Check (FBT-FACT; 30). Fidelity was also self-rated by the therapists and the child’s parents immediately after each session. Again, therapists were not trained in fidelity rating in order to maintain a real-world stance. Each therapist audio-recorded at least one case and submitted session 1, 2, and 3 from phase 1, plus one additional session from phase 1, two sessions from phase 2, and one session from phase 3 for fidelity rating. FBT fidelity item responses are on a 7-point Likert scale from ‘1’ (‘not at all’) to ‘7’ (‘very much’), with higher scores indicative of greater use of the FBT components in a given session.

### Data analyses

#### Item-level analyses

Inter-observer reliability was calculated for each item from the first three sessions of FBT using the intraclass correlation coefficient (ICC [30];). Two-way mixed ICC were used. Cicchetti [31] classified the magnitude of ICC scores as follows; below 0.40 = poor, 0.40 to 0.59 = fair, 0.60 to 0.74 = good, and 0.75 to 1.00 = excellent. We focused our fidelity analysis primarily on session 1, 2 and 3 with the ICC data. The reason for this is that other data suggests fidelity within these sessions is sufficient for predicting end of treatment outcomes [19, 20].

#### Scale-level analyses

One-way repeated measures analysis of variance (ANOVA) was used to examine mean session scores from sessions 1, 2, and 3, as well as one additional Phase 1 session, and two Phase 2 (Phase 2a and Phase 2b) sessions across raters. Not enough ratings were available to complete this analysis for Phase 3. Post hoc pairwise comparisons were completed with Bonferroni correction. Significance was set at *p* < 0.05.

## Results

### Demographics

Eight therapists (six female, two male) participated in this study from four different sites. Four therapists were social workers, two were psychologists, and two were psychiatrists. Therapists ranged in age from 28 years to 60 years and had worked in eating disorder services for an average of 7.4 years (range was one month to 14 years).

### Item-level analyses

Mean scores on items from session 1,2 and 3 indicated trends toward higher fidelity ratings by parents and therapists and lower ratings by the peer rater, compared to the expert rater (Table 1). Intraclass correlation coefficients revealed that agreement was the best between expert and peer, with excellent, good, or fair agreement in 7 of 13 items (Table 2). There were only four such values when comparing expert to parent ratings, and two such values comparing expert to therapist ratings. The remainder of the ICC values indicated poor agreement.

**Table 1:** Mean FBT fidelity rating score (and standard deviation) per item by expert, therapist, parent and peer rater from session 1, 2 and 3. FBT fidelity scale ranges from 1= “not at all” to 7 = “very much”

	Expert	Therapist	Parent	Peer
Session 1 - Greet Family	4.62 (1.41)	5.12 (1.46)	6.75 (0.46)	3.63 (2.13)
Session 1 - Take history	5.62 (1.60)	5.37 (0.92)	6.50 (1.04)	3.50 (1.60)
Session 1 - Externalize	4.87 (1.55)	6.00 (1.20)	6.87 (0.35)	3.87 (2.10)
Session 1 - Intense scene	4.75 (1.58)	5.50 (1.60)	6.62 (0.58)	3.50 (2.20)
Session 1 - Charge parents	5.12 (1.25)	6.12 (0.99)	6.81 (0.53)	3.75 (1.83)
Session 2 - Take history around food	5.12 (1.25)	5.86 (1.35)	6.93 (0.18)	3.87 (0.83)
Session 2 - One more bite	5.00 (1.31)	6.00 (1.15)	6.87 (0.35)	3.12 (1.96)
Session 2 - Align patient with Siblings	5.00 (1.41)	5.83 (1.47)	6.71 (0.93)	3.12 (1.64)
Session 3 - Focus discussion on food	5.25 (1.39)	5.33 (1.51)	6.83 (0.26)	4.62 (1.41)
Session 3 - Help parental dyad with refeeding	5.37 (1.30)	4.83 (2.32)	6.67 (0.41)	4.37 (1.19)
Session 3 - Siblings efforts	4.50 (1.07)	5.33 (1.97)	6.50 (0.63)	3.00 (2.20)
Session 3 - Modify criticism	5.00 (1.31)	4.67 (1.51)	6.92 (1.28)	2.87 (1.46)
Session 3 - Distinguish interests from AN	5.62 (0.74)	5.83 (1.17)	7.00 (0.00)	4.50 (1.41)

**Table 2:**
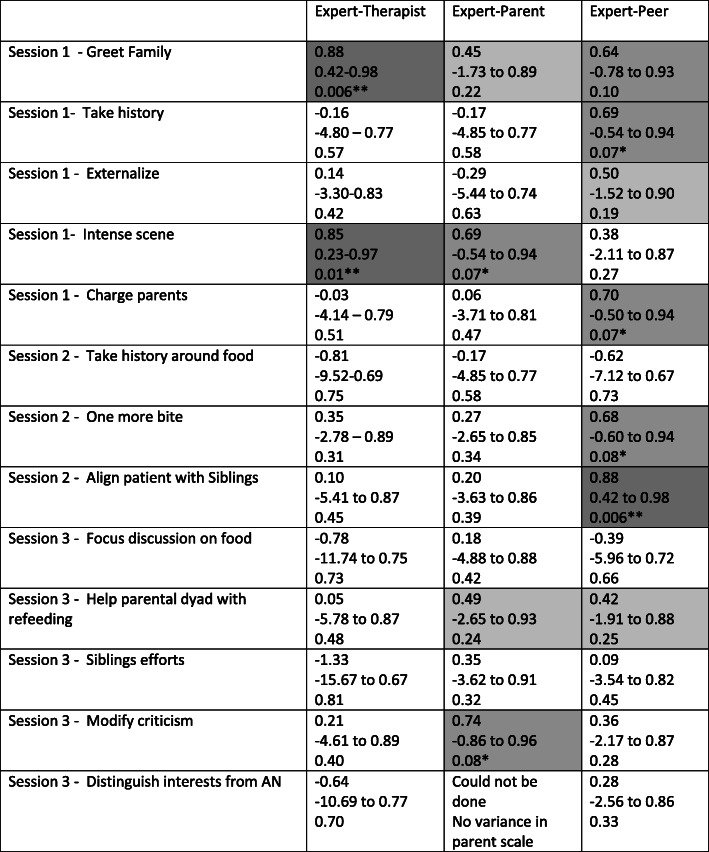
Item level comparisons on FBT fidelity scale from sessions 1, 2 and 3 using Intra class Correlation Coefficients (ICC) values are displayed with confidence intervals and *p* value

** Darkest gray = excellent, medium gray = good, light gray = fair, no shading = poor agreement

### Scale-level analyses

One-way repeated measures ANOVA indicated significant differences in the mean fidelity ratings of all sessions evaluated (Table 3). Phase 3 could not be tested due to insufficient data. Post hoc pairwise comparisons indicated that parents often rated higher fidelity to FBT compared to the peer rater, but also compared to the expert rater and therapist rater as well (Table 3). On the additional phase 1 session, the expert also rated higher than the peer (*p* < 0.049), and in session 2 the therapist rater rated significantly higher than the peer rater (*p* < 0.045). There were no significant differences between expert and therapist scores.

**Table 3:** Scale level comparison with repeated measures ANOVA. Mean session scores (and standard deviation) by expert, therapist, parent and peer rater. FBT fidelity scale ranges from 1= “not at all” to 7 = “very much”

	Expert	Therapist	Parent	Peer	F	Sig	Partial eta Squared	Pairwise Comparison
Session 1 *n* = 8	5.00 (1.22)	5.62 (1.10)	6.71 (0.42)	3.65 (1.81)	13.4 (3.21)	0.001	0.66	Parent > Expert, *p* < 0.016; Parent > Therapist, *p* < 0.025; Parent > Peer, *p* < 0.008
Session 2 *n* = 6	5.22 (1.13)	5.83 (1.35)	6.86 (0.16)	3.78 (1.28)	10.9 (3.15)	0.001	0.69	Therapist > Peer, *p* < 0.045; Parent > Peer, *p* < 0.012
Session 3 *n* = 6	5.23 (1.12)	5.20 (1.56)	6.78 (0.42)	4.33 (1.06)	4.96 (3.15)	0.014	0.50	Parent > Expert, *p* < 0.039; Parent > Peer, *p* < 0.023
Phase 1 Session *n* = 6	5.67 (1.58)	5.07 (1.09)	6.77 (0.36)	3.17 (1.45)	11.2 (3.15)	0.001	0.69	Expert > Peer, *p* < 0.049; Parent > Peer, *p* < 0.019
Phase 2a Session *n* = 5	5.40 (0.82)	5.20 (1.57)	6.47 (0.69)	2.93 (0.92)	8.84 (3.12)	0.002	0.69	Parent > Peer, *p* < 0.034
Phase 2b session *n* = 6	5.25 (1.78)	5.19 (1.62)	6.50 (0.47)	3.25 (1.39)	6.20 (3.15)	0.006	0.55	Parent > Peer, *p* < 0.024
Phase 3 session *n* = 1	7.0	6.33	6.17	3.33	No statistical testing possible			

## Discussion

This is the first study to examine fidelity to FBT from the perspectives of multiple raters including expert, therapist, parent, and peer. Although these data are exploratory in nature, the peer rater demonstrated the highest concordance with the expert rater, while therapist raters and parent raters demonstrated low concordance with the expert when looking at items from session 1, 2 and 3. Scale level analysis indicated that expert fidelity ratings for phase 1 treatment sessions scores were significantly higher than the peer ratings and, that parent fidelity ratings tended to be significantly higher than the other raters across all three treatment phases. Interestingly, there were no significant differences between the mean therapist ratings and the expert ratings. The item level data suggest that peers might present the best method for fidelity assessment, whereas the scale level analysis indicates that therapist ratings do not differ significantly from expert ratings, and therefore therapist self-rating may be an acceptable option for fidelity rating in these types of settings. Of course, therapist ratings would be much more cost efficient.

One possible reason for poor agreement in rating is that parents, therapists and the peer did not receive training in fidelity rating. This was intentional, as we had hoped to see how fidelity rating would occur in real-world clinical settings and how raters would compare to the expert. Although the peer rater was not a novice in the field of FBT, she had not received any formal training in FBT session fidelity rating. There also seemed to be a pattern of the greatest agreement in session 1 which is the most prescriptive, and lower levels of agreement as treatment progressed. It is likely easier to rate a session which has several well-described interventions. We theorize that rating may become more difficult and more divergent as the treatment becomes less prescriptive. This pattern has been seen in other studies as well [19, 20]. In addition, peer ratings were significantly lower compared to the expert on the scale level analysis. We postulate that peers may be more vigilant and critical with respect to treatment interventions, whereas an expert may be more accepting of variations on intervention delivery as they have more experience in this regard.

It is also difficult to determine which analysis is more helpful in rating fidelity; the item level or scale level analysis. In a laboratory setting in which interrater agreement is critical on each item, and training is received in fidelity rating, the item level analysis may be most appropriate. The broader approach of scale level analysis might be most helpful when looking at significant differences rather than agreement. However, when learning an intervention such as this in the real-world, it is important to achieve acceptable levels of fidelity on all key interventions of the treatment (all items). This is where a threshold approach could be considered with respect to examining treatment fidelity. What level of fidelity is good enough? This question remains to be answered in terms of the level of fidelity related to treatment outcomes. Without this data it is difficult to determine what cut-off is adequate. Our previous study [25] used an a priori threshold of 80% (5.6/7) as adequate fidelity on each session (as opposed to each item), as this level is considered “considerable”, however, how this threshold relates to outcome is unknown. In this previous study, only one therapist out of eight met this bar, whereas the mean scores of the all eight therapists where actually quite similar to fidelity ratings in other FBT trials [20, 32], suggesting that perhaps this threshold is too high. In fact, a recent study by Dimitropoulos and colleagues [19] suggests that 4/7 or higher (set a priori) is adequate fidelity. However, this study found that adherence was not related to outcome in terms of percent ideal body weight [19], emphasizing the complexity of how fidelity and adherence relate to outcome. Further work is needed in this area.

Some of our findings align with Chapman and colleagues [21] who reported that youth and caregiver fidelity raters for adherence to a substance abuse treatment protocol for adolescents were highly inaccurate compared to treatment experts. Therapists and trained raters were generally consistent with ratings of treatment experts [21]. The trained raters were bachelor to masters level research assistants with no experience in delivering the treatment. Parents and youth were much more likely to indiscriminately endorse the occurrence of key treatment components [21].

Also, somewhat in alignment with our findings, the reliability of therapist self-report has been called into question in prior research. Modest to weak agreement between therapist and observer fidelity ratings have been demonstrated in two studies of motivational interviewing with adults [23, 24]. These raters were trained coders, most with a masters’ degree and experience in treatment delivery. An additional study of treatment fidelity for children with conduct problems reported that trained observers (research students pursuing a masters’ degree in social work, marriage and family therapy, or psychology) rated less frequent use of evidence based interventions compared to therapists’ fidelity ratings [22].

Interestingly, the degree of interrater agreement between the fidelity ratings of therapists and observers may vary depending on the type of treatment being delivered, as seen in a study by Hogue and colleagues [33]. These researchers examined the fidelity of evidence-based practices for adolescent behaviour problems by comparing therapist to observer fidelity ratings. Observers were trained observational raters and had a masters’ degree in social work or psychology. Findings showed that therapists providing a family therapy intervention consistently provided fidelity ratings that aligned with those of expert raters. However, when these therapists rated their fidelity for motivational interviewing along with cognitive behaviour treatment, their inter-rater concordance with expert ratings was poor [33]. The underlying mechanisms for this difference in alignment based on intervention remain unclear [33].

Innovative methods to capture and rate fidelity are currently being studied. Caperton and colleagues [34] examined “thin slices” of motivational interviewing sessions in order to determine the shortest session fragment for which fidelity by interrater agreement was similar between the thin slice and the full session. The thin slices were captured at random. These authors determined that approximately one third of a session, or about 9 min, had sufficient agreement to approach interrater levels for the full session. Although these authors caution that these results apply to motivational interviewing only, this could be a valid method to be studied for FBT to determine what length of session would be sufficient to be rated for fidelity. There may be unique features to FBT that would not be captured by this method, for example charging the parents with the task of refeeding, which only occurs at the end of session one.

As an alternate measure of fidelity, innovative ways of measuring therapist *competence* in FBT are also being developed. Lock and colleagues [35] recently used a new method to evaluate therapists’ behavioural skills acquisition of FBT using online training. Forty-six therapists were randomized to receive regular online training or enhanced FBT online training with extra modules focused on the two key elements of agnosticism and externalization. These authors recorded participants’ responses to video vignettes and then rated them on competence using a newly developed measure. Although this method does not examine fidelity directly, it is an innovative method of evaluating therapists’ acquisition of these skills. The findings indicated that the group receiving advanced training performed better in terms of skill in the area of agnosticism.

There are several important limitations to this study. The first limitation is the small sample size and the exploratory nature of the data. Future studies should aim to include more therapists, peers and experts. Caution should be used in interpreting our findings as our small sample size was used in statistical testing. In addition, there may have been bias present in the way the fidelity data were collected. For example, parents knew that the therapist was collecting the data and sending it to the researchers. Thus, parents may have felt pressure to provide inflated scores, as they would not want their scores to negatively affect their relationship with their therapist. Although efforts were made to ensure that parent reports were confidential and would be faxed directly to the researchers without being seen by the therapists, parents may still have been subject to this bias. It is important to note that the ICC data are based on 13 items from the first three sessions of FBT. It is quite possible that later sessions of Phase 1, along with Phase 2 and 3 would have produced different results, and that the level of agreement may depend on the phase of treatment. However, evidence suggests that fidelity within these sessions is sufficient for predicting end of treatment outcomes [19, 20], and that early weight gain by the start of session 4 predicts with 80% probability end of treatment remission [36, 37]. Thus, we felt fidelity to these first three sessions was likely the most important and could be a way to assess fidelity efficiently in clinical practice settings.

Although concordance was highest between the peer and expert rater in our study using ICC data at the item level, it is important to note that no significant differences were seen on mean session scores between the expert and the therapists. Therefore, when thinking of the most pragmatic and cost-efficient method of fidelity measurement, therapist rating would likely meet these requirements. However, an important caveat to our findings is that even when a pragmatic measure of treatment fidelity is identified, some authors have found that it is difficult for practitioners to sustain fidelity assessment in the long-term, unless an organizational culture is present that recognizes fidelity assessment as essential for optimal clinical outcomes [8]. Therapist drift can occur over time, and thus, ongoing attention to fidelity is important well after a new treatment has been implemented and maintained.

## Conclusions

In summary, FBT fidelity ratings conducted by a peer rater demonstrated the highest concordance with those of the expert rater at the item level, and on a scale level, no significant differences were seen between therapists and the expert rater. The lower concordance of therapists’ self-ratings and parents’ ratings with the expert ratings at the item level may be due to the complex interplay of relationships, and other factors. Rating treatment fidelity objectively may be compromised when one is receiving or delivering the treatment. Although the peer rater demonstrated highest concordance with the expert, therapists themselves may provide the most pragmatic and cost-effective method of fidelity assessment. There remains a need to develop innovative and efficient means of rating FBT fidelity. Rating smaller segments of sessions (thin slices) or using indirect methods of capturing therapist competence may offer potential solutions and should be investigated in future research.

## Data Availability

The datasets during and/or analysed during the current study available from the corresponding author on reasonable request.

## Abbreviations

AN: Anorexia nervosa
BN: Bulimia nervosa
EBT: Evidence-Based Treatment
FBT: Family Based Treatment
ICC: Intraclass Correlation Coefficient
